# Tradeoffs between air pollution mitigation and meteorological response in India

**DOI:** 10.1038/s41598-020-71607-5

**Published:** 2020-09-09

**Authors:** Abhishek Upadhyay, Sagnik Dey, Sourangsu Chowdhury, Rajesh Kumar, Pramila Goyal

**Affiliations:** 1 Centre for Atmospheric Sciences, Indian Institute of Technology Delhi, New Delhi, India; 2 Centre of Excellence for Research on Clean Air, IIT Delhi, New Delhi, India; 3 School of Public Policy, IIT Delhi, New Delhi, India; 4 National Center for Atmospheric Research, Boulder, CO USA; 5 Max Planck Institute for Chemistry, Mainz, Germany

**Keywords:** Climate sciences, Environmental sciences, Environmental social sciences

## Abstract

To curb the staggering health burden attributed to air pollution, the sustainable solution for India would be to reduce emissions in future. Here we project ambient fine particulate matter (PM_2.5_) exposure in India for the year 2030 under two contrasting air pollution emission pathways for two different climate scenarios based on Representative Concentration Pathways (RCP4.5 and RCP8.5). All-India average PM_2.5_ is expected to increase from 41.4 ± 26.5 μg m^−3^ in 2010 to 61.1 ± 40.8 and 58.2 ± 37.5 μg m^−3^ in 2030 under RCP8.5 and RCP4.5 scenarios, respectively if India follows the current legislation (baseline) emission pathway. In contrast, ambient PM_2.5_ in 2030 would be 40.2 ± 27.5 (for RCP8.5) and 39.2 ± 25.4 (for RCP4.5) μg m^−3^ following the short-lived climate pollutant (SLCP) mitigation emission pathway. We find that the lower PM_2.5_ in the mitigation pathway (34.2% and 32.6%, respectively for RCP8.5 and RCP4.5 relative to the baseline emission pathway) would come at a cost of 0.3–0.5 °C additional warming due to the direct impact of aerosols. The premature mortality burden attributable to ambient PM_2.5_ exposure is expected to rise from 2010 to 2030, but 381,790 (5–95% confidence interval, CI 275,620–514,600) deaths can be averted following the mitigation emission pathway relative to the baseline emission pathway. Therefore, we conclude that given the expected large health benefit, the mitigation emission pathway is a reasonable tradeoff for India despite the meteorological response. However, India needs to act more aggressively as the World Health Organization (WHO) annual air quality guideline (10 µg m^−3^) would remain far off.

## Introduction

Exposure to air pollution poses a serious health burden globally^1^. In India, 670,000 (95% CI 550,000–790,000) deaths and 0.9 years (0.8–1.1) life expectancy loss were attributable to ambient air pollution exposure in 2017^2^ making it the second-largest health risk factor after maternal and child malnutrition^2^. In addition to mortality, air pollution exposure in India has been associated with child growth failure^3^, low birthweight^4^, and multiple non-communicable diseases amongst children and adults^2,5^. Ambient PM_2.5_ exposure has increased substantially in the past from 1990 to 2015 in India^1^ and is expected to increase in the near future under global warming^6,7^.

Realizing the staggering burden of air pollution exposure in the entire country, the Government of India has launched National Clean Air Program (NCAP) in early 2019 with a target of 20–30% reduction in ambient PM_2.5_ in the non-attainment cities by 2024. Recent studies^8^ have shown that emissions from household activities contribute ~ 30% to ambient PM_2.5_ and therefore the recent policy of distributing clean cooking fuel through Pradhan Mantri Ujjwala Yojana (PMYU) to 80 million households seems to be a plausible pathway to reduce ambient PM_2.5_. Estimates suggest that complete mitigation of emissions from household sources could have potentially helped India in achieving its annual national ambient air quality standard (NAAQS) of 40 μg m^−3^ for the base year 2015^9^. However, this is not enough for the National Capital Region (NCR) including Delhi to meet the NAAQS and also, other outdoor sources are expected to increase in the future due to growing population and its energy demand. Therefore, we need to define alternate emission pathways for India which are feasible to implement and can facilitate India to improve its good air quality in the near future.

**Figure 1:**
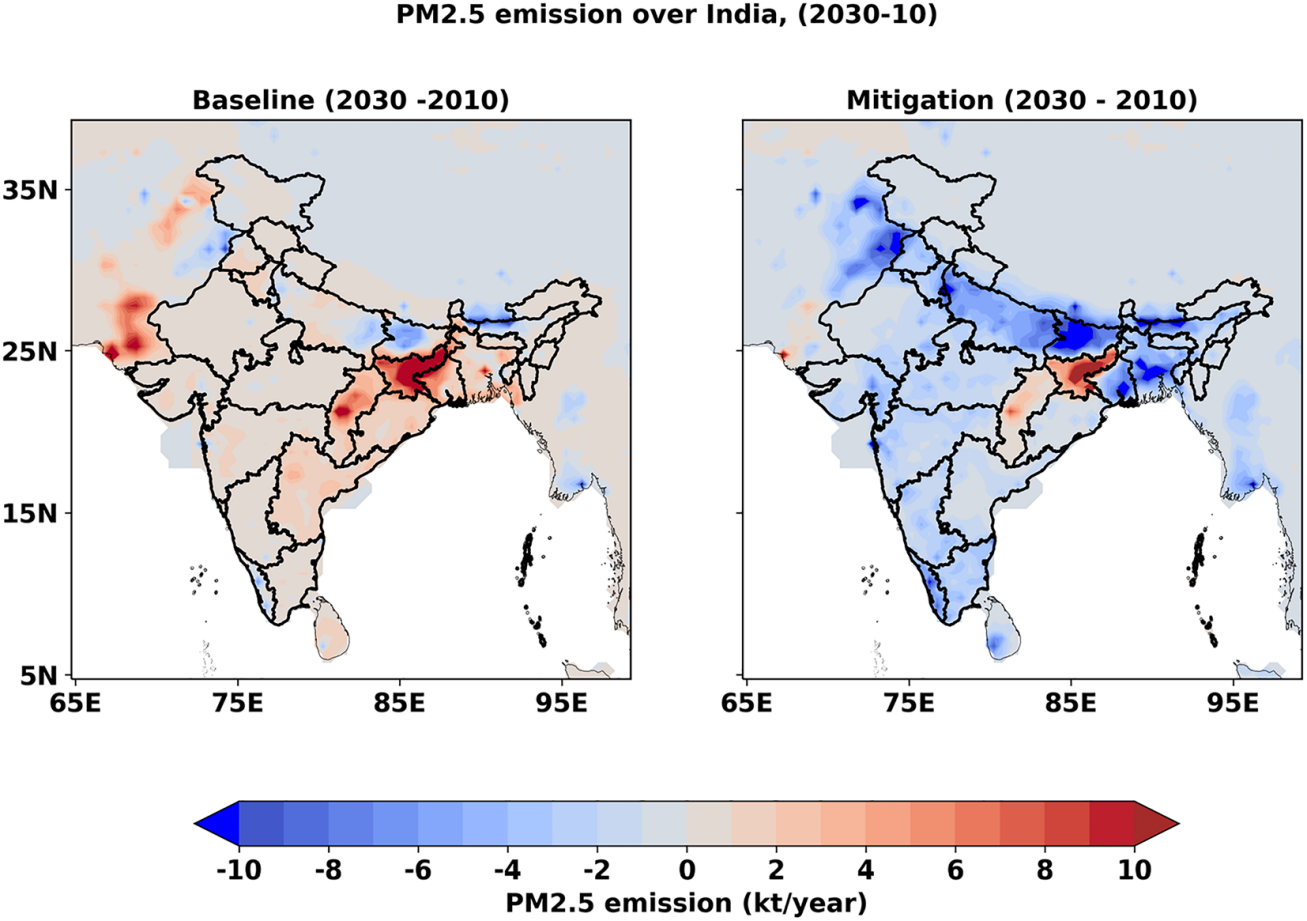
Projected changes in primary PM_2.5_ emission (kt year^−1^) from anthropogenic sources in 2030 relative to 2010 in the Indian subcontinent for the (**a**) baseline and (**b**) mitigation emission pathways. The maps are generated in Python.

In this study, we explored two emission pathways developed under Evaluating the Climate and Air Quality Impact of Short-Lived Pollutants (ECLIPSE) emission inventory developed by International Institute for Applied Systems Analysis (IIASA)^10^. Current legislation emission pathway (hereafter baseline emission) considers current environmental laws with known implementation delay and it assumes complete enforcement of existing legislation in the near future. Mitigation emission pathway has been developed to reduce short-lived climate pollutants (SLCP) like black carbon (BC) to get climate benefits along with air quality improvement. These mitigation measures of targeted species typically reduce emissions of other co-emitted species at the same time^10,11^. In the baseline emission pathway, total primary PM_2.5_ emission over the Indian subcontinent is expected to increase by 17.4% from 8,190 kt year^−1^ in 2010 to 9,620 kt year^−1^ in 2030 (Fig. 1) with a large increase expected in the states of Jharkhand, Chhattisgarh, Odisha, Andhra Pradesh, and Telangana (see Fig. S1 for the state boundaries). On the contrary, primary PM_2.5_ emission is expected to reduce by 43.3% in 2030 from 2010 over the domain following the mitigation emission pathway, where all the states except Jharkhand and Chhattisgarh (dominated by mining activities and associated industries) show a large decrease. BC and organic carbon (OC) emissions over the domain are expected to reduce by 3.3% and 4.6% in 2030 relative to 2010 in the baseline emission pathway, while large reduction of 77.8% and 69.7% is expected in BC and OC emissions respectively, following the mitigation emission pathway in 2030 (Fig. S2). SO_2_ emissions over the domain are expected to almost double and reach ~ 19,400 kt year^−1^ in 2030 in both emission pathways, whereas NO_x_ emission is expected to increase by 66.8% and 30.3% in baseline and mitigation emission pathway respectively in 2030 from 8,224 kt year^−1^ in 2010 (Fig. S2). This is primarily due to a larger contribution from the industry and energy sector in the near future with continued reliance on coal.

We used a regional chemical transport model known as the Weather Research and Forecasting model coupled with Chemistry (WRF-Chem) in climate mode to simulate PM_2.5_ for the baseline year 2010 using ECLIPSE emission inventory for 2010 over the Indian subcontinent, and then project the same for these two emission pathways for the year 2030 under RCP4.5 and RCP8.5 scenarios (see “Methods”). The two emission pathways have different BC, OC, other particulate matter, SO_2_, NOx, NMVOC and NH_3_ emissions over the model domain, whereas major greenhouse gases (GHGs) are identical in the two pathways and only vary across the RCP scenarios (Table S1 in SI). The model performance for the baseline year 2010 has been extensively evaluated in our previous work^12^. We estimate the mortality burden attributable to ambient PM_2.5_ using the current demographic and epidemiological information for 2010 following the GBD (Global Burden of Disease) approach^2^ and project the same for 2030 using the projected population and baseline mortality for the two emission pathways. We further examine the impacts of the projected emission pathways on the regional meteorology in 2030 and discuss the tradeoff between air pollution mitigation and meteorological response to aerosol-radiation feedback through direct effect.

## Results

### Ambient PM_2.5_ projection

**Figure 2:**
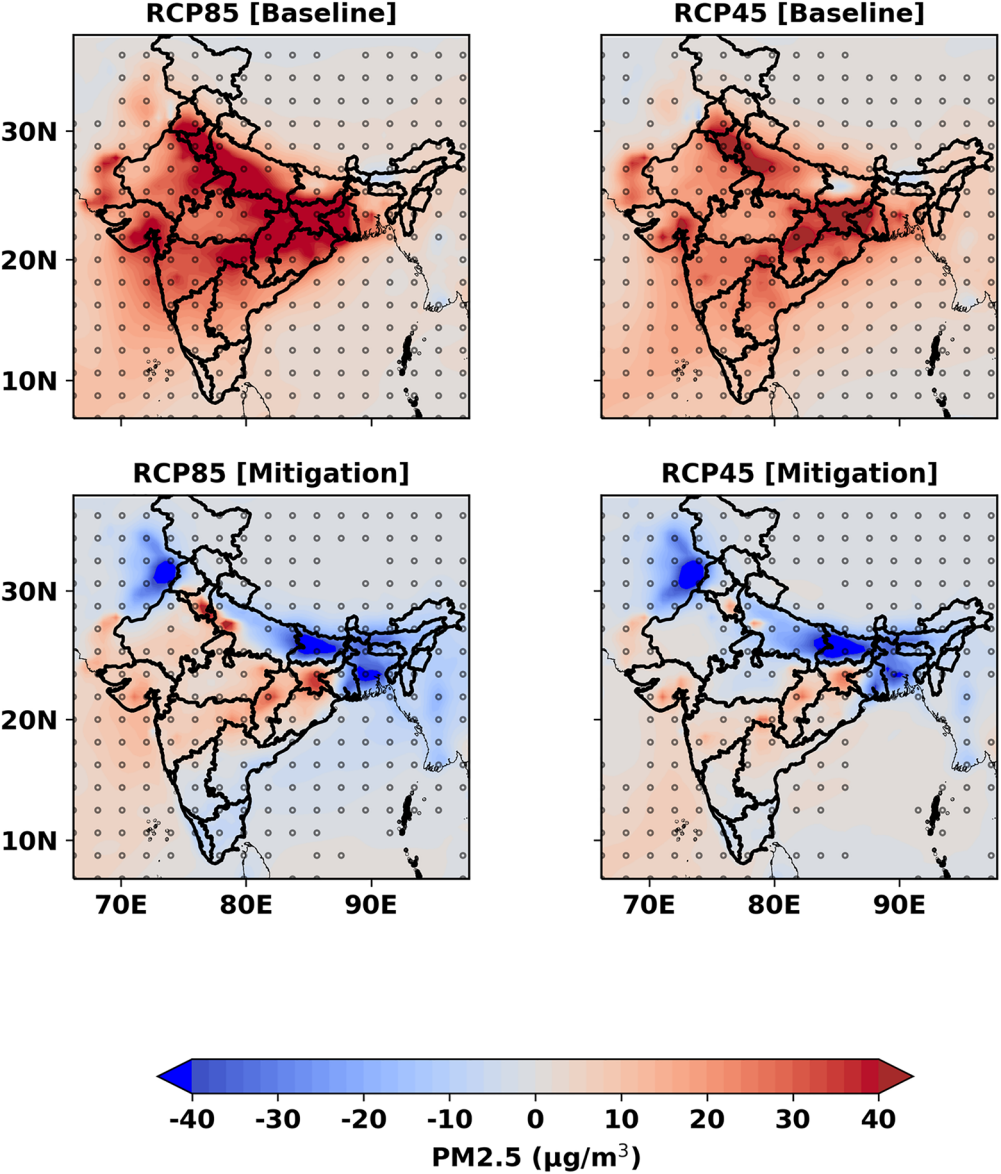
Projected changes in ambient PM_2.5_ concentration in 2030 relative to 2010 in the Indian subcontinent for the (top panel) baseline and (bottom panel) mitigation emission pathways under the (left) RCP8.5 and (right) RCP4.5 scenarios. Dots are representing a 95% level of statistical significance. The maps are generated in Python.

Changes in ambient PM_2.5_ distribution over India in 2030 with the two contrasting emission pathways (baseline and mitigation) under RCP8.5 and RCP4.5 scenarios are shown in Fig. 2. Following the baseline emission pathway, all-India average (± 1σ) ambient PM_2.5_ is expected to increase by 47.6% from 41.4 ± 26.5 μg m^−3^ in 2010 to 61.1 ± 40.8 μg m^−3^ under the RCP8.5 scenario and by 40.6% to 58.2 ± 37.5 μg m^−3^ under RCP4.5 scenario in 2030. A larger rise in PM_2.5_ concentration (~ 30–40 µg m^−3^) is projected in the north and central India compared to south India (~ 10–20 µg m^−3^). This implies that air quality would worsen in the near future even if the ongoing legislations are applied with full efficiency as assumed in this emission pathway. On the other hand, with the proposed mitigation emission pathway that focusses on reducing SLCP, the all-India average (± 1σ) ambient PM_2.5_ is projected to slightly reduce (by 2.9%) to 40.2 ± 27.5 µg m^−3^ in 2030 compared to the baseline year 2010 under RCP8.5 scenario and by 5.3% to 39.2 ± 25.4 µg m^−3^ under RCP4.5 scenario. A substantial reduction in PM_2.5_ in most of the Indo-Gangetic Basin (IGB) except in Delhi, surrounding satellite towns, the states of Jharkhand and Chhattisgarh and some parts of central India (see Fig. S1 for the geographical distributions of the states) is the reflection of the emission reduction mostly from the residential sector under the mitigation emission pathway, which is the single largest contributor to ambient PM_2.5_ pollution in India^13,14^. However, it is noteworthy that Delhi and its surrounding region would remain out of attainment. Ambient PM_2.5_ is expected to increase here by more than 30 µg m^−3^ under the RCP8.5 scenario, and by a slightly lower margin (10–20 µg m^−3^) under the RCP4.5 scenario.

### Meteorological response

**Figure 3:**
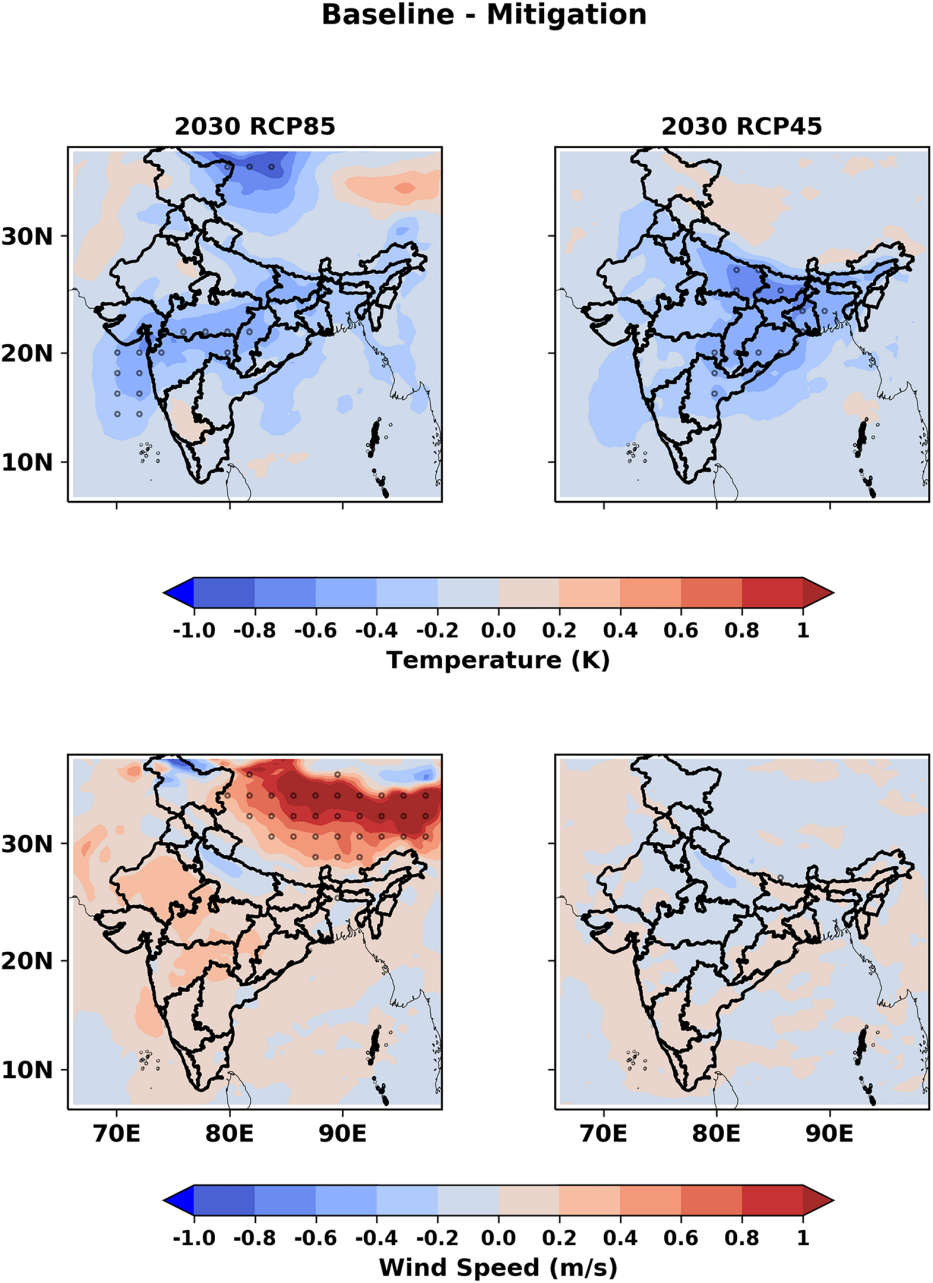
Differences (baseline-mitigation) in projected (top panel) 2 m air temperature and (bottom panel) wind speed between the baseline and mitigation emission pathways in 2030 over the Indian subcontinent under the (left panel) RCP8.5 and (right panel) RCP4.5 scenario. Dots are representing a 95% level of statistical significance. The maps are generated in Python.

The simulated 2 m-air temperature in the mitigation emission pathway is higher than the baseline emission pathway for both RCP8.5 and RCP4.5 scenarios as shown by the negative (i.e. baseline-mitigation) differences (Fig. 3). Aerosols scatter and/or absorb solar radiation and allow less solar insolation reaching the surface (known as dimming) resulting in a decrease in temperature^15,16^. Therefore, the larger reduction in aerosol concentration in the mitigation emission pathway (as discussed in the previous section) compared to the baseline emission pathway is expected to enhance the temperature in both RCP scenarios in 2030. However, the differences in mean temperature are not significant in most of the grids as shown by the 95% confidence level of statistical significance (Fig. 3). Under RCP8.5 scenario, central India is expected to warm significantly higher in the mitigation emission pathway than in the baseline emission pathway, while the eastern IGB (see Fig. S1) seem to be more affected under RCP4.5 scenario in the mitigation emission pathway. We note that aerosols can influence radiation budget through indirect and semi-direct effect too, but this experiment only considers the direct impact of aerosols. Also, the transport of pollution from outside the model domain may contribute to the observed difference between the baseline and mitigation emission pathway. The contribution of lateral boundary conditions to BC mass concentrations in India is less than 5%^17^. So, we anticipate this difference to be smaller relative to the direct impact of aerosols within the domain. Overall, the mean 2 m temperature over the Indian landmass is higher by 0.20 ± 0.16 K and 0.22 ± 0.18 K for the mitigation emission pathway compared to the baseline emission pathway following RCP8.5 and RCP4.5 scenario, respectively. In the case of wind, we observe a mixed response to the changing pollution load, but the projected differences are not statistically significant over the Indian landmass (Fig. 3). We only expect a significant increase in wind speed over the Tibetan Plateau under the RCP8.5 scenario in the mitigation emission pathway relative to the baseline emission pathway. Since the natural aerosol (both dust and sea salt) loading depends on wind speed, no significant enhancement in natural PM_2.5_ is expected over the Indian landmass in the mitigation emission relative to the baseline emission pathway in 2030. For this reason, we attribute projected changes in future PM_2.5_ primarily to anthropogenic activities. Overall, therefore, the mitigation emission pathway seems to be a rationale choice for India in the near future.

### Expected health benefit of choosing the mitigating emission over the baseline emission pathway

**Figure 4:**
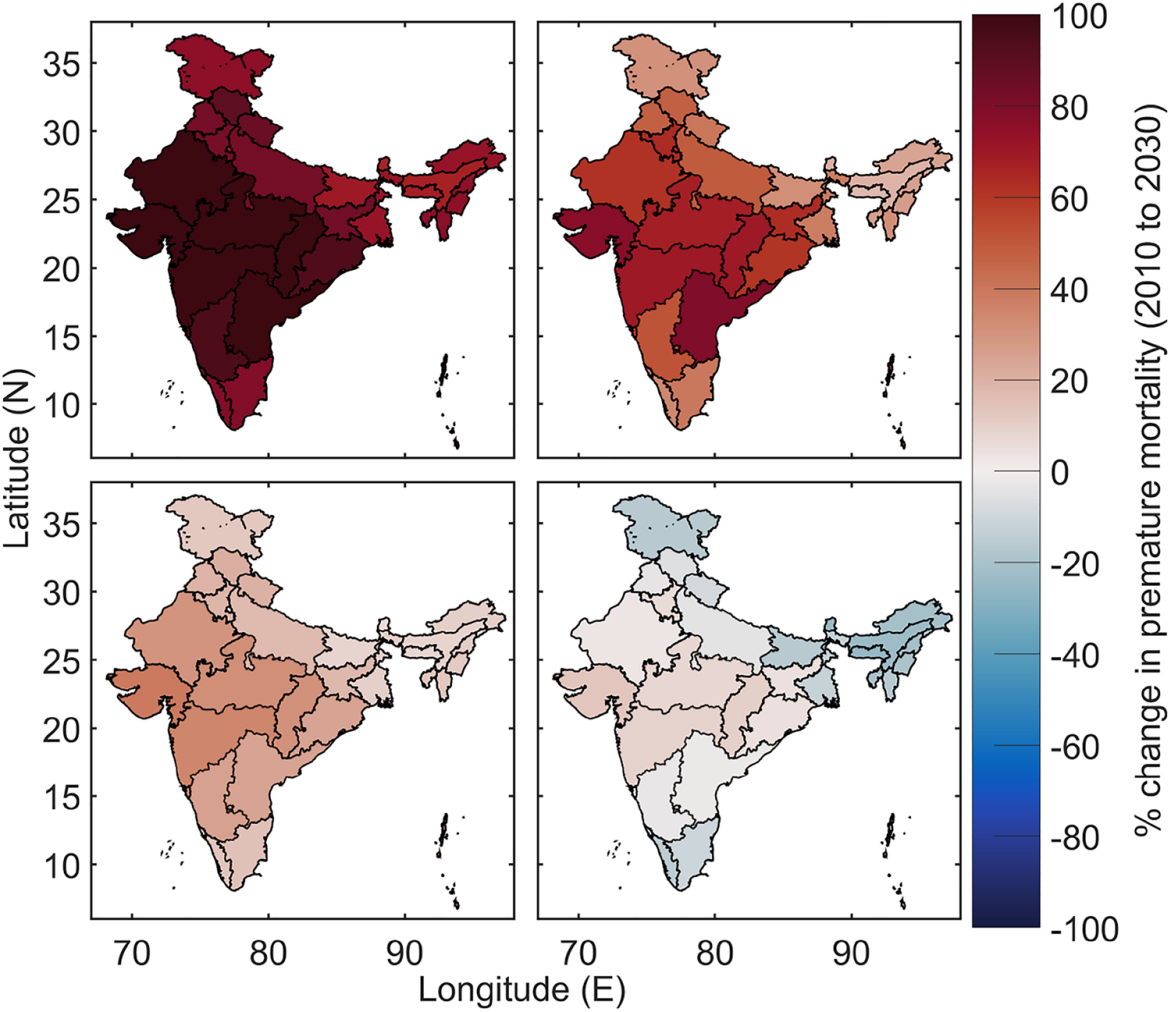
(Upper panel) Percentage changes in premature mortality burden attributed to ambient PM_2.5_ exposure over India in 2030 considering projected population and baseline mortality with the (left) baseline emission and (right) mitigation emission pathways under the RCP8.5 scenario relative to the baseline year 2010. (Lower panel) Percentage changes in premature mortality burden attributed to ambient PM_2.5_ over India in 2030 considering population and baseline mortality similar to 2010 and with the (left) baseline emission and (right) mitigation emission pathways under the RCP8.5 scenario relative to the baseline year 2010. The maps are generated in Matlab R2018.

Since the projected ambient PM_2.5_ concentrations in 2030 are not significantly different for RCP8.5 and RCP4.5 scenarios (Fig. 2), we used only RCP8.5 scenarios to estimate the health burden. Estimates of mortality burden using global exposure mortality model (GEMM) relative risk functions (see “Methods”) show that ambient PM_2.5_ was responsible for 1,050,340 (5–95th CI 866,510–1,240,410) deaths in 2010 over India. The mortality burden is expected to rise by 88.7% (62–118%) to 1,982,370 (1,408,040–2,706,900) in 2030 under RCP8.5 scenario with the baseline emission pathway (Fig. 4). If India switches to the mitigation emission pathway in the near future, the mortality burden attributable to ambient PM_2.5_ is expected to increase in 2030 by 52.4% (30–76%) to 1,600,580 (1,132,420–2,192,300) relative to the baseline year 2010.

**Figure 5:**
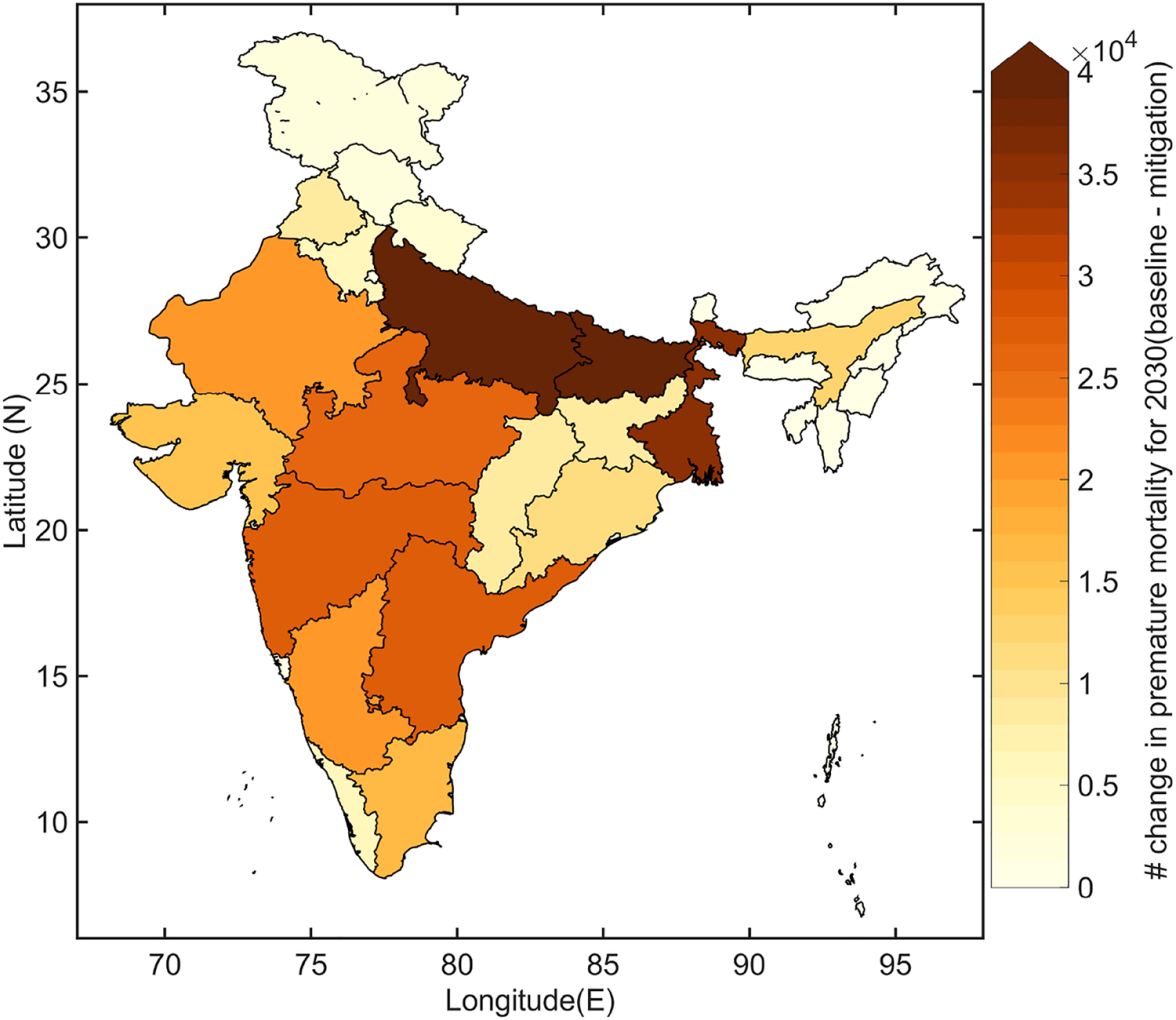
Averted mortality burden attributable to ambient PM_2.5_ exposure over India in 2030 due to the mitigation emission pathway relative to the baseline emission pathway under the RCP8.5 scenario. The maps are generated in Matlab R2018.

We note that estimated changes in mortality burden not only depends on the expected rise in ambient PM_2.5_ exposure in 2030 relative to 2010 (as shown in Fig. 2) but also depends on the projected increase in exposed population (Fig. S3), a shift in the age structure (Fig. S4) and changes in baseline mortality rates (Fig. S5). In 2030, the population is projected to increase by more than 45% in all the states relative to 2010 with a larger increase in the population aged over 60 years. The relative risks of the non-communicable diseases (NCDs) and lower respiratory infection (LRI) are higher for the aged population^18^ and hence the age shift along with the population rise is expected to increase the premature mortality burden in 2030 even if the exposure levels remain unchanged (i.e. the mitigation emission pathway case). When the exposure is also projected to increase in tandem (as in the baseline emission pathway case), the premature mortality burden would be higher. However, the baseline mortality rates for adult NCDs and LRI are projected to decrease in 2030 compared to 2010 (Figs. S5 and S6) due to an envisaged improvement in the health infrastructure and healthcare, which would arrest the increase in premature mortality burden to some extent. To isolate the impact of changing PM_2.5_ on the premature mortality burden from the other socio-demographic factors, we carry out a sensitivity study (see “Methods”). We find that the mortality burden is expected to increase by 20% (− 0.3–41%) to 1,260,900 (1,046,900–1,480,500) in 2030 due to only the changes in PM_2.5_ exposure in the baseline emission pathway (Fig. 5). In the mitigation emission pathway, the premature mortality burden is expected to decrease marginally by 3% (− 20–15%) to 1,018,400 (839,360–1,203,600) in 2030.

Our results suggest that choosing the mitigation emission pathway over the baseline emission pathway in the near future is expected to avert 381,790 (275,620–514,600) deaths in 2030 (Fig. 5), out of which 242,500 (63.8% of the total averted deaths) averted deaths can be attributed only to the changes in PM_2.5_ exposure and the remaining 36.2% to the changes in socio-demographic changes. The state-level burden estimates are compiled in Table S1 (see SI). A large health benefit is expected in the three populated states in the IGB—Uttar Pradesh (74,916 averted deaths), Bihar (39,941 averted deaths) and West Bengal (35,338 averted deaths). On the other hand, only 5% of deaths could be averted in Delhi in 2030 by following the mitigation emission pathway over the baseline emission pathway. Overall at a national scale, choice of mitigation emission pathway would at least contain ambient PM_2.5_ exposure in 2030 similar to the level in 2010 and this would advert 19.3% of expected death.

## Discussion and conclusions

Ambient PM_2.5_ exposure and resulting health burden are very high in India. In this work, we explored two contrasting emission pathways for India using the WRF-Chem model to examine their suitability in containing the rising air pollution problem in the near future. We found that the choice of mitigation emission pathway in the Indian subcontinent would contain ambient PM_2.5_ exposure in India in 2030 under RCP8.5 and RCP4.5 similar to the 2010 level, while it is expected to increase by 47.6% and 40.6% under RCP8.5 and RCP4.5 scenarios respectively in the baseline emission pathway. We understand that the magnitudes of the impacts may deviate from the results presented here if the neighboring countries (within the domain) do not follow the same pathways; however, since India’s contribution to the overall emissions is the largest in this region, we believe that our results will hold. Furthermore, the reduction of air pollution is not expected to impact the wind speed significantly in near future, which implies that the production of natural aerosols (that depends on wind speed) would not be enhanced and hence the total ambient PM_2.5_ trend would follow the trend of anthropogenic PM_2.5_.

We further found that the better air quality in the mitigation emission pathway relative to the baseline emission pathway would come at a cost of 0.3–0.5 °C additional warming. This is consistent with a recent study^19^ which showed that reducing aerosol emissions from anthropogenic sources would contribute additional warming. Though statistically, this temperature rise is not significant everywhere, it may impact tropospheric chemistry and consequently ozone pollution in the near future, at least in those local hotspots. Given that, few recent studies^6,20^ have suggested that ozone formation is expected to be enhanced in higher temperature, such issue needs to be addressed separately. Higher ozone and additional warming in the mitigation scenario may reduce crop productivity^21^; however, this may be partially compensated by a lower ambient PM_2.5_ that also has a negative association with productivity^22^. The additional warming may enhance heat stress condition; however, this can be tackled by an effective heat management plan that has been proven to save lives in India^23^. Quantifying such impact for the two emission pathways is beyond the scope of this work and will be reported separately.

In both emission pathways, the mortality burden is expected to increase in 2030 compared to 2010 primarily due to rise in population and age shift towards older ages (partially compensated by an expected improvement in baseline mortality), but 19.3% deaths can be avoided in the mitigation emission pathway because of much lower ambient PM_2.5_ exposure compared to the baseline emission pathway. Furthermore, following the mitigation emission pathway has an additional benefit for India because the monsoon precipitation may be enhanced by mitigating SLCPs as interpreted by Shindell et al.^24^. Therefore, because of the expected health benefit, we feel that the mitigation emission pathway is a good tradeoff relative to the baseline emission pathway in the near future despite the additional warming.

We also note that even following the mitigation emission pathway is not enough for India to achieve the WHO annual AQG of 10 µg m^−3^ (as the pollution level is projected to be ~ 4 times the WHO-AQG over most of India). This implies that India needs to be more aggressive in its battle against air pollution to reduce the mortality burden attributable to air pollution so that it can meet the sustainable development goals set by the WHO. We note that India has already launched several policy measures in recent years in that direction. The NCAP and PMUY are expected to reduce ambient PM_2.5_ in the urban and rural area. Besides, India has also launched national carbonaceous aerosol program^25^ to explore climate-air pollution co-benefits from emission mitigation. This work will guide in exploring potential benefits of these recent efforts.

## Methods

### Model set up

The Weather Research Forecasting model coupled with chemistry (WRF-Chem) simulates meteorological and chemical parameters of the atmosphere simultaneously^26^. Version 3.6 of this model is compiled in climate mode for this work (CWRF). CWRF is the modified version of WRF with the ability to assimilate annually varying mixing ratio for five greenhouse gases CO_2_, N_2_O, CH_4_, CFC-11 and CFC-12 from an input file CAMtr_volume_mixing_ratio (see Table S1). This enables radiation parameterization in the model to respond to changes in greenhouse gas concentrations^6,27^. The model has been set up over India to predict ambient PM_2.5_ concentration over India for present-day (the year 2010) and future (the year 2030). These simulations are performed over single domain at a resolution of 50 km × 50 km over the entire Indian region. The model domain is centered at 22.5° N and 82.5° E with 79 grids in both South-North and West–East directions [domain co-ordinates, 4.35 N°–38.55° N and 63.75° E–101.24° E] and 30 vertical levels with 50 hPa pressure at the top.

Physics and chemistry schemes are selected based on previous studies over this domain. Parameterization of various form of water is done through single moment microphysics scheme Purdue Lin^28^. It includes a mixed-phase process suitable for fine resolution simulation. RRTMG (Rapid Radiative Transfer Model for General circulation model)^29^ radiation scheme is used for shortwave and longwave radiation processes. Yonsei University PBL scheme^30^ is used for planetary boundary layer processes. Land surface physics is represented by NOAH land surface scheme in the setup. Bulk aerosol module GOCART (Goddard Chemistry Aerosol Radiation and Transport)^31^ has been used along with RADM2 (Regional acid deposition model)^32^ gas-phase chemistry. GOCART is a bulk aerosol module, which divides BC and OC into hydrophilic and hydrophobic form and considers other primary particulate matter and sulphate as secondary aerosols. It does not include secondary organic aerosols, leading to an underestimation in aerosol load. Dust and sea-salt are treated in five and four size ranges, respectively. The model setup includes dry deposition of gas and aerosol species and sub-grid wet scavenging. Aerosol optical properties are calculated based on the volume approximation.

### Meteorological input

NCAR CESM global bias-corrected output has been used as model input to generate the initial and boundary conditions for meteorological fields. This bias-corrected data is available in three RCP scenario 8.5, 6 and 4.5 to support WRF and MAPS (https://rda.ucar.edu/datasets/ds316.1/). The data sets have been generated using version 1 of the CESM model also known as CESM1 and then bias-corrected against global atmospheric and surface European Centre for Medium-Range Weather Forecasting (ECMWF) Interim Reanalysis (ERAI). Data files are available for two different timeframe 20th-century simulation which is for the year 1951 to 2005 and simulation with three RCP future scenario for years 2006–2100. More details regarding this data set are available in NCAR technical note^33^.

### Chemical boundary condition

The chemical boundary condition for the model has been obtained from CAM-Chem model simulations for different RCP scenarios^34^. This has been used for both initial and lateral boundary conditions for chemical species through MOZBC preprocessor. This global model output is available at a spatial resolution of 1.9° NS × 2.5° WE for each month. The data has been archived at the NCAR High-Performance Storage System (HPSS) and can be requested from NCAR. CAM-Chem simulations are driven by RCP4.5 and RCP8.5 scenarios, where the difference between ECLIPSE and RCP air pollution emission scenarios can have some effect on our simulations. However, we anticipate these differences to be less than 10% for two reasons. First, we performed the simulations for the whole year and the effect of initial conditions decrease rapidly to less than 10% in about 48 h in regional models^35^. Second, Kumar et al.^17^ showed that lateral boundary conditions contribute less than 5% to BC levels over India. Since BC and other aerosols have similar lifetimes, we expect a similar contribution of lateral boundary conditions for other aerosols as well.

### Emission inventory

ECLIPSE project was carried out at IIASA, Austria with the target of mitigating climate change and simultaneously improving air quality. Integrated assessment model Greenhouse gas Air pollution INteractions and Synergies (GAINS) has been used to create ECLIPSE emission dataset that includes emissions of long-lived greenhouse gas and shorter-lived species in consistent framework^36^. Version 5a of ECLIPSE global emission has been used in this work, this provides emission data for SO_2_, NO_x_, CO, VOC, NH_3_, PM_10_, PM_2.5_, BC and OC for various emission scenario at 0.5° × 0.5° spatial resolution (https://www.iiasa.ac.at/web/home/research/researchPrograms/air/ECLIPSEv5.html). Choice of the baseline and mitigation emission pathways is explored in this work to examine the impacts of mitigation strategies against the ongoing policies. The baseline scenario is the current legislation scenario that considers current environmental law with known implementation delay and it assumes complete enforced implementation of existing legislation in future. Mitigation scenario has been developed to reduce the SLCPs to get climate benefits along with air quality improvement. This climate optimized SLCP emission reduction measures are grouped into three categories (a) measures that affect emissions of CH_4_, (b) technical measures to reduce BC, (c) non-technical measures to reduce BC. These mitigation measures of one species typically affect several species at the same time^10,11^. Even though CH_4_ is considered as a greenhouse gas in Kyoto protocol with a lifetime of 9 ± 1 years, development of SLCP mitigation scenario referred to it as an SLCP because of its relatively smaller lifetime compared to CO_2_^10^. Impact of mitigation emission pathway is seen for primary PM_2.5_, gaseous precursors, BC and OC emissions (Figs. 1 and S2). Emissions for these three species PM_2.5_, BC and OC are projected to decrease by 43.2%, 77.8% and 69.7% respectively. While mitigation emission pathway is not effective for SO_2_, as SO_2_ emission is the same with baseline and mitigation pathway in the year 2030. NOx emission is projected to increase with a higher rate of 66.8% in 2030 compared to 2010 in baseline emission pathway whereas this increasing pace is comparatively lower (30.3%) in mitigation emission pathway. Biogenic emission is online and is estimated based on land use using Guenther scheme in the model^37^, which depends on the temperature and photosynthesis rate. We have used similar land use in all the sensitivity experiments in this study. Open biomass burning emission is taken from MODIS fire data.

### Model validation

The model has been validated over India previously by various researchers including us^11,12^. The model simulated ambient PM_2.5_ showed statistically significant correlation with satellite-derived and ground-based PM_2.5_, but the model has a low bias. More details about the model validation are provided in the SI. Nonetheless, we believe that the results are meaningful as the bias in the present-day and near-future simulations would not be very different.

### Estimates of mortality burden

We estimated mortality attributed to ambient PM_2.5_ exposure (rounded off to nearest 10 s) in 2010 and projected PM_2.5_ exposure in the year 2030 following the baseline and mitigation emission pathway under RCP8.5 scenario for all NCDs and LRI for the exposed adult population (age > 25 years) using the relation from our previous studies^13,38^. We did not find significant differences in estimated ambient PM_2.5_ under RCP4.5 and RCP8.5 scenario; hence health estimates are carried out only for the RCP8.5 scenario. We used the GEMM^18^ to calculate the age-dependent hazard ratios for NCD + LRI among adults (age > 25 years). The GEMM, unlike the earlier integrated exposure–response functions^39^, does not rely on data on household air pollution exposure, second-hand smoking and active tobacco smoking to compromise for the higher end of ambient PM_2.5_ exposure. It was built by incorporating 41 epidemiological studies on ambient air pollution performed across 16 countries which also includes a study involving Chinese men with long term ambient PM_2.5_ exposure up to 84 µg m^−3^^18^. The GEMM provides hazard ratios for NCD + LRI at 5-year interval age classes.

The adult population at 5-year age intervals was obtained from the Census of India 2011 for estimating mortality from NCDs + LRI for 2010. For 2030, we use the gridded age classified population under SSP2 (middle of the road) scenario^7,40^. Age distribution across the exposed population and change in population is shown in Figs. S3 and S4, respectively. We obtain the baseline mortality rates for NCD and LRI from the GBD estimates for 2010 and 2030 (Reference scenario). The GBD Foresight study (https://vizhub.healthdata.org/gbd-foresight/) provides baseline mortality at the national level, we distribute the baseline mortality rates across the states by keeping the uniform distribution of disease incidence as of 2010. We also estimate the mortality burden for 2030 assuming the population (and its age distribution) and baseline mortality of 2010 and exposure of 2030 (under the baseline and mitigation emission pathways). The difference in these estimates relative to the estimates with exposure, population and baseline mortality of 2030 would segregate the impact of changing PM_2.5_ exposure on the mortality burden from the other socio-demographic factors.

### Assumptions and uncertainty analysis

In this study, we assume several things to derive the results and interpret them. The model performance for the baseline year 2010 has been extensively evaluated in our previous work^12,13^. Spatial distributions of anthropogenic PM_2.5_ and carbonaceous aerosols are matching well with satellite-derived and reanalysis products^12^. Predicted concentrations are slightly higher with ECLIPSE emission inventory compared to EDGAR-HTAP emissions.

We assume that the GEMM hazard ratios for exposure to ambient air pollution and the spatial heterogeneity in baseline mortality rates hold for 2030. We note that we did not estimate uncertainty in our burden estimates as there are no data to validate the projected PM_2.5_ exposure in 2030. The mortality calculations in our study are accompanied by 95% CI. The error in the coefficients of the GEMM hazard ratio was used to estimate the 95% CI values, which distributed log-normally, and 1,000 random draws of the hazard ratios were selected for an estimate of exposure in each state. Similarly, the baseline mortality estimates (central value with 95% CI) were distributed log-normally and 1,000 random draws were selected for each state. Therefore, from 1,000 × 1,000 (1,000,000) estimates of mortality obtained for a PM_2.5_ exposure for a state, the mean (95% CI) are presented here.

## Supplementary information

Supplementary Infrormation.

## Data Availability

All data will be made available on request from the first author.
