# Supplementary material for: Tradeoffs between air pollution mitigation and meteorological response in India
Source: Sci Rep. 2020 Sep 9;10:14796. doi: 10.1038/s41598-020-71607-5 (PMC7481194; doi:10.1038/s41598-020-71607-5)
Supplement: Supplementary file 1 — Supplementary Infrormation. [file 41598_2020_71607_MOESM1_ESM.pdf]

Supplementary material

Tradeoffs between air pollution mitigation and meteorological response in India

[1]Abhishek Upadhyay, [1,2,3]Sagnik Dey*, [1,5]Sourangsu Chowdhury, [4]Rajesh Kumar, [1]Pramila Goyal

[1]Centre for Atmospheric Sciences, Indian Institute of Technology Delhi, New Delhi, India
[2]Centre of Excellence for Research on Clean Air, IIT Delhi, New Delhi, India
[3]School of Public Policy, IIT Delhi, New Delhi, India
[4]National Center for Atmospheric Research, Boulder, Colorado, USA
[5]Max Planck Institute for Chemistry, Mainz, Germany.

*Corresponding author: sagnik@cas.iitd.ac.in

The SI contains seven figures, two tables and one section.

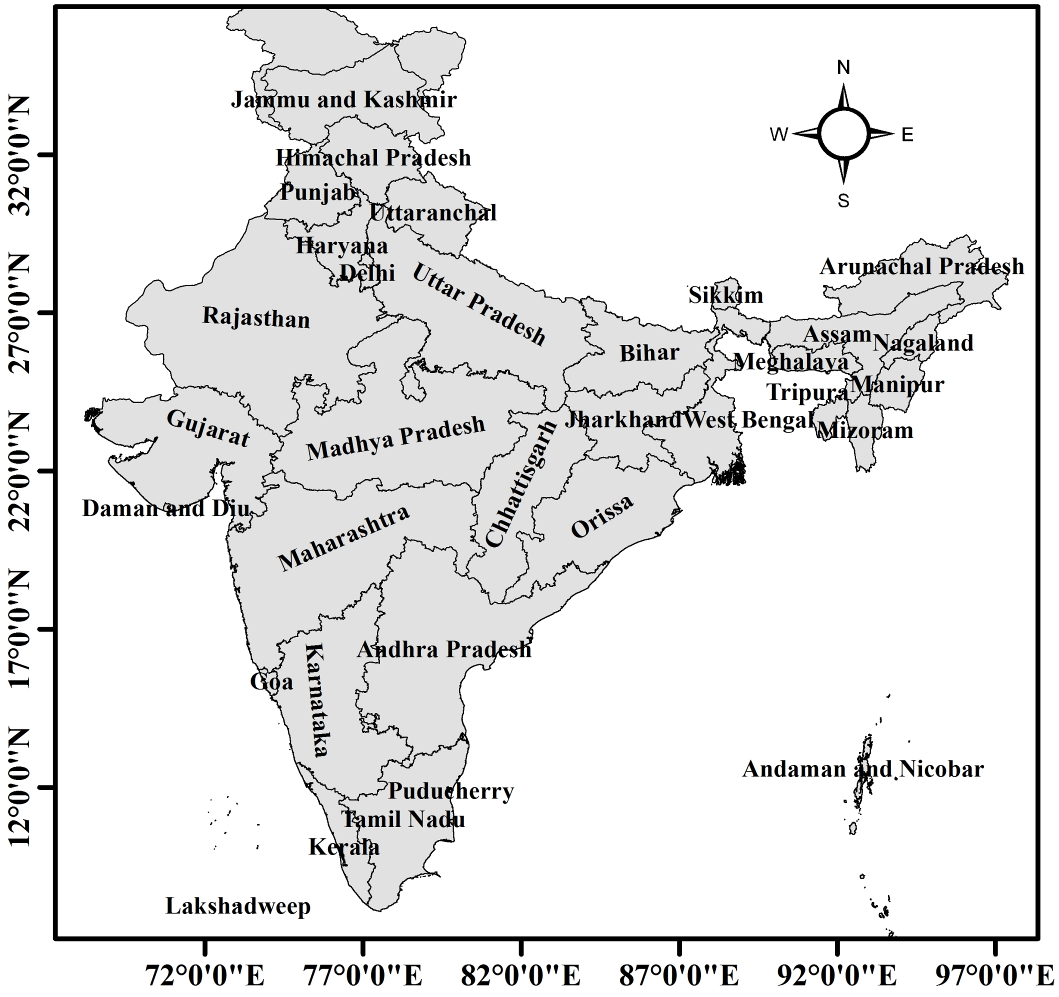

Figure S1. Political map of India shows the administrating boundaries of the states and UTs. The results in this work are summarized and discussed in terms of the geographical positions of the states. The Indo-Gangetic Basin (IGB) covers the states of Punjab, Haryana, Delhi, Uttar Pradesh, Bihar, Jharkhand and West Bengal. The map is generated in QGIS V3.14.1.

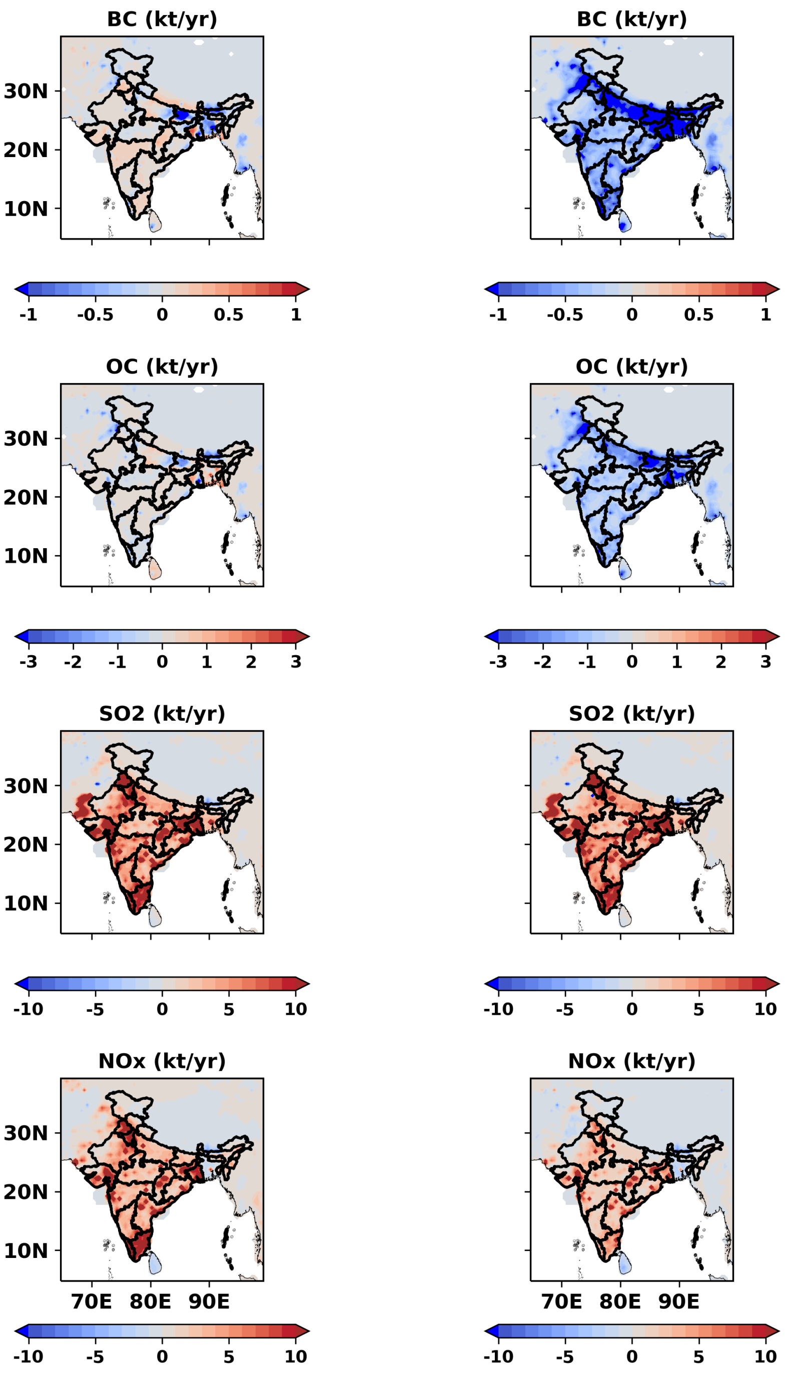

Figure S2. Changes in emission (2030 - 2010) for BC, OC, SO_2 and NOx represented in kt/year over India in year 2030 following the (left panel) baseline emission pathway and (right panel) mitigation emission pathway compared to year 2010 over India. The maps are generated in Python.

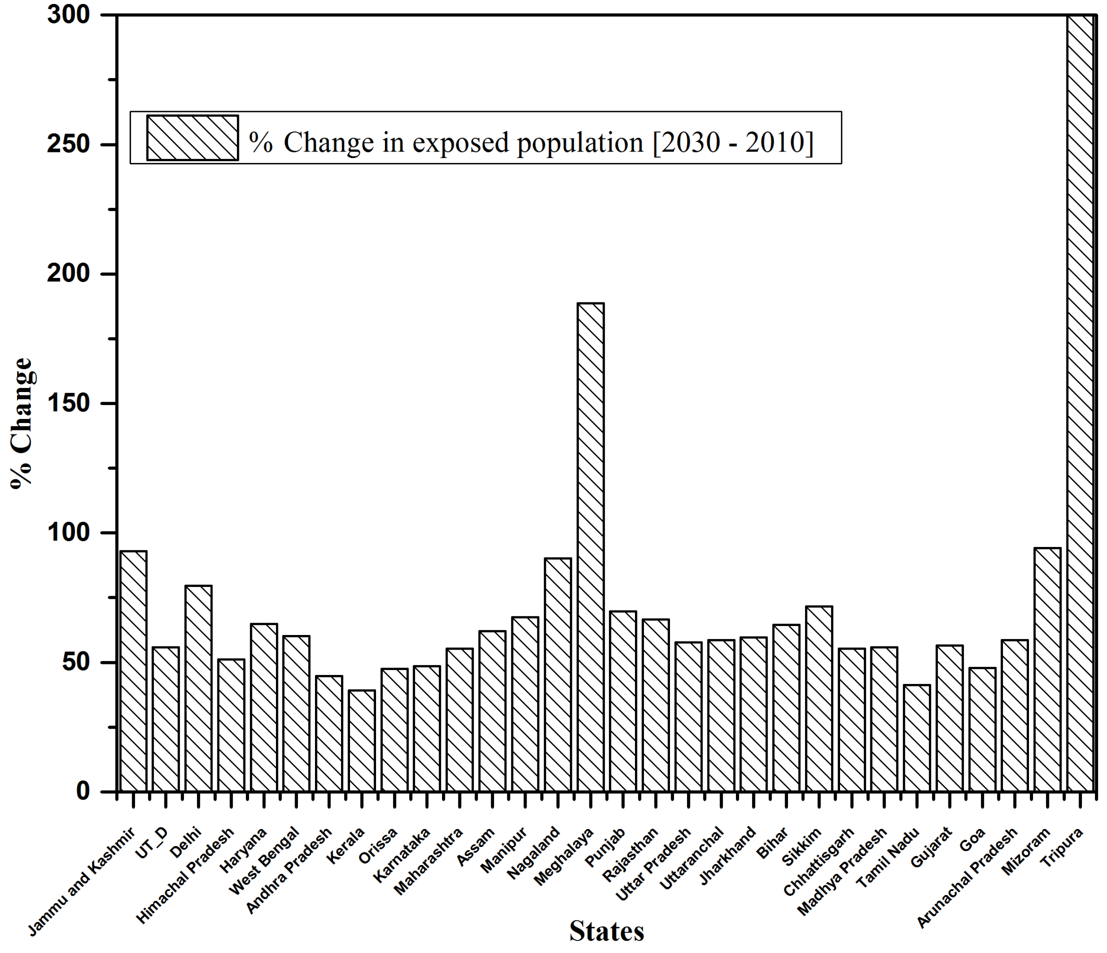

Figure S3. Percentage changes in exposed population (for age more than 25 years) in the year 2030 compared to the baseline year 2010 for each state of India. UT_D means all other UTs except Delhi.

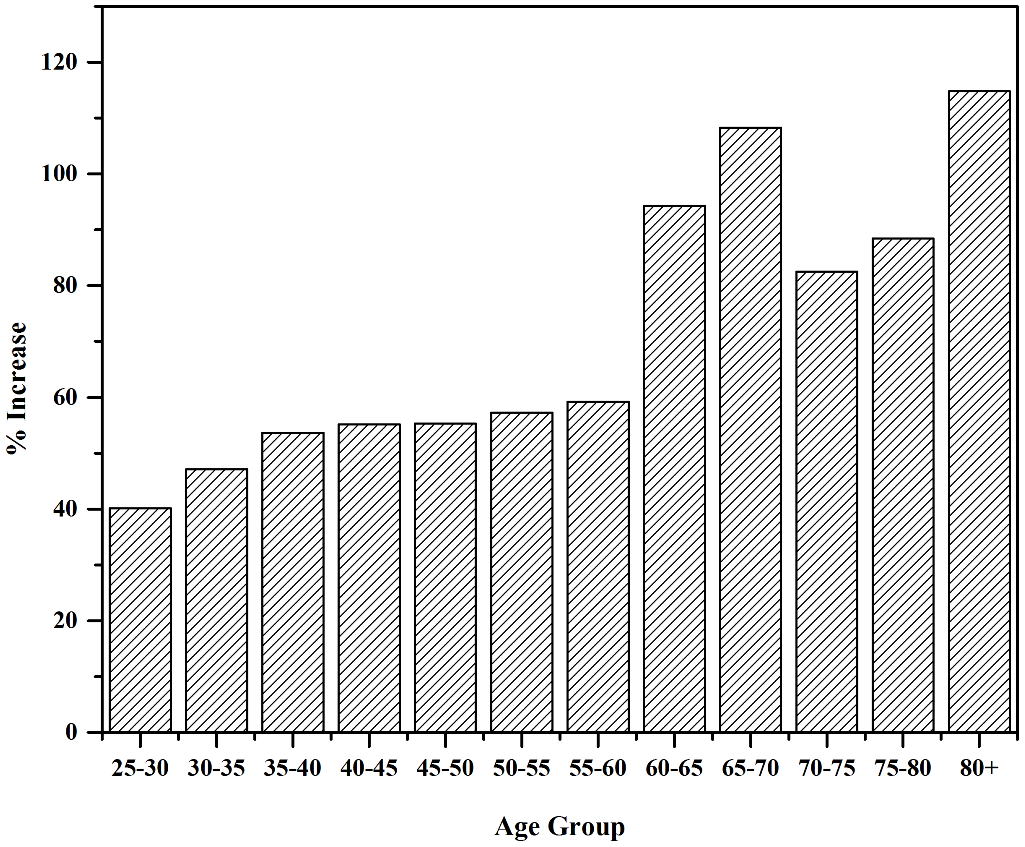

Figure S4. Percentage change in exposed population (for age more than 25 years) at every 5-year age intervals for 2030 compared to the baseline year 2010.

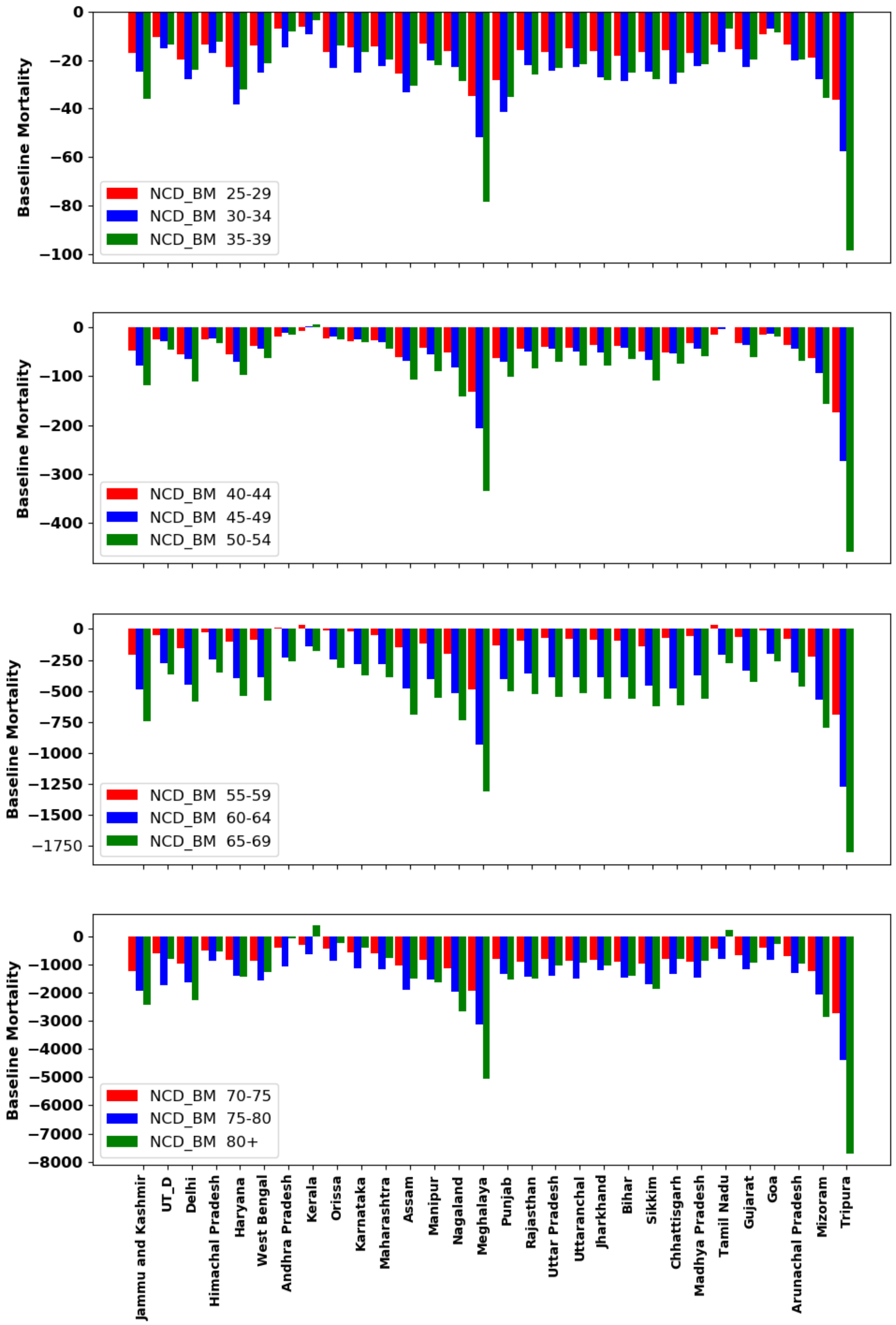

Figure S5. Percentage changes in age-specific baseline mortality (BM) values for adult non-communicable diseases (NCDs) at state level in 2030 relative to 2010 at the respective age ranges shown by the numbers (e.g. NCD_BM 80+ represents NCD baseline mortality for the population aged more than 80 years).

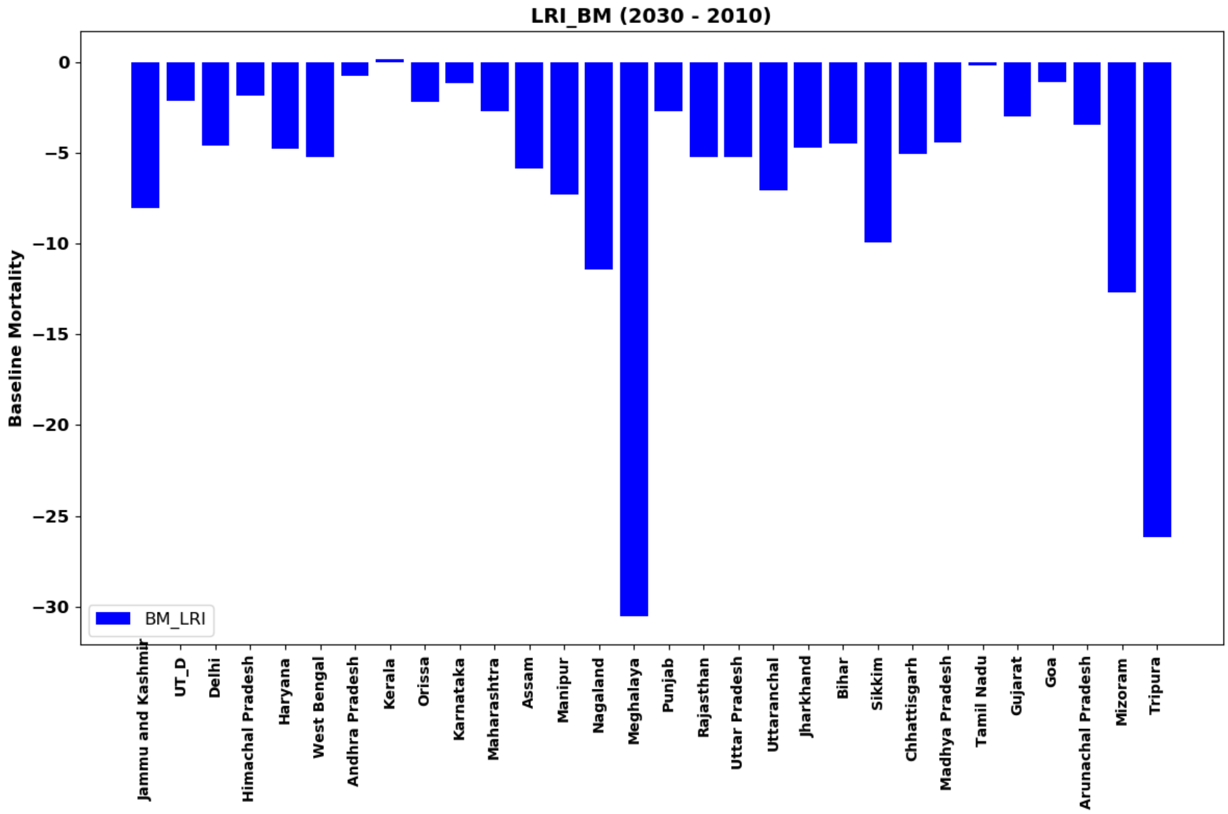

Figure S6. Percentage changes in baseline mortality (BM) for adult lower respiratory infection (LRI) at state level in 2030 relative to 2010.

Model Validation. In our previous study, WRF-Chem simulation has been evaluated for $PM_{2.5}$ and carbonaceous aerosols (BC and OC) over India against different data sets[1,2]. WRF-Chem setup in this study are almost similar to the previous studies, except inclusion of climate mode in the current study. Previously, we found that the model-simulated ambient $PM_{2.5}$ showed 0.87 and 0.9 correlation coefficient and 8.4 and 10.2 µg m^{-3} RMSE with EDGAR and ECLIPSE emission inventory, respectively[1]. The validation was carried out with $PM_{2.5}$ data from CPCB site at Delhi for year 2010 and satellite-derived $PM_{2.5}$ database from van Donkelaar et al[3]. This dataset is developed with multiple satellite product and includes multiple observation for bias correction (details are available in the literature). In another study, Bran and Srivastava[4] showed that the model under-predicts ambient $PM_{2.5}$ in the winter, although the correlation coefficient between model simulated and measured $PM_{2.5}$ is 0.81 (significant at 98% CI). Conibear et al.[5] reported that the normalized mean biases during the winter, spring, summer and autumn seasons are -0.24, -0.07, 0.69 and -0.10, respectively. Since this simulation is carried out in climate mode, we compared WRF-Chem simulation for the year 2010 with ECLISE emission with van Donkelaar et al.[3] database (Fig. S7). The spatial patters suggest that the model over-predicted the ambient $PM_{2.5}$ in the eastern Indo-Gangetic Plain and part of Peninsular India, while it under-predicted over the arid region in the western India. We also found negligible difference between RCP4.5 and RCP8.5 scenario. It is not possible to validate future projection, but we expect the biases to be of similar magnitude since the parameterization schemes are same. We interpret our results keeping these biases in mind.

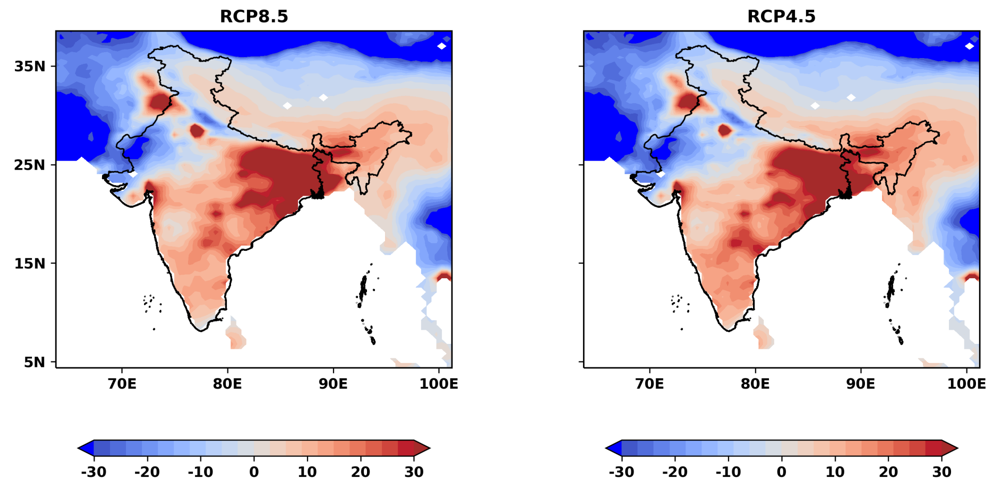

Figure S7. Difference (in µg m^{-3}) between simulated ambient $PM_{2.5}$ under (a) RCP8.5 and (b) RCP4.5 scenario with ECLIPSE emission and satellite derived ambient $PM_{2.5}$ over India for year 2010. The maps are generated in Python.

References

1. Upadhyay, A., Dey, S. & Goyal, P. A comparative assessment of regional representativeness of EDGAR and ECLIPSE emission inventories for air quality studies in India. *Atmos. Environ.* 117182 (2019) doi:https://doi.org/10.1016/j.atmosenv.2019.117182.

2. Upadhyay, A., Dey, S., Chowdhury, S. & Goyal, P. Expected health bene fi ts from mitigation of emissions from major anthropogenic PM 2 . 5 sources in India : Statistics at state level *. *Environ. Pollut.* **242**, 1817–1826 (2018).

3. Donkelaar, A. Van *et al.* Global Estimates of Fine Particulate Matter using a Combined Geophysical-Statistical Method with Information from Satellites , Models , and Monitors. (2016) doi:10.1021/acs.est.5b05833.

4. Bran, S. H., & Srivastava, R. Investigation of $PM_{2.5}$ mass concentration over India using a regional climate model. *Environ. Pollut.* 224: 484-493 (2017).

5. Conibear, L., Butt, E. W., Knote, C., Arnold, S. A., & Spracklen, D. V. Residential energy use emissions dominate health impacts from exposure to ambient particulate matter in India. *Nat. Comm.* 9: 617 (2018).

Table S1. Concentrations of the GHGs used in the simulation. Note that the GHG concentrations are identical in the baseline and mitigation emission pathways.

Year	Species (unit)	RCP4.5	RCP8.5
2010	CO_2 (ppmv)	389.1	389.3
	N_2O (ppbv)	322.9	323.0
	CH_4 (ppbv)	1767.0	1778.6
	CFC11 (ppbv)	337.8	337.8
	CFC12 (ppbv)	524.9	524.9
2030	CO_2 (ppmv)	435.0	448.0
	N_2O (ppbv)	337.1	341.9
	CH_4 (ppbv)	1829.9	2132.0
	CFC11 (ppbv)	248.6	248.1
	CFC12 (ppbv)	436.8	436.3

Table S2. Mortality (with 95% CI is in the second row) attributable to $PM_{2.5}$ exposure for states and union territories (UTs) of India in the base year 2010 and the year 2030 using the baseline and mitigation emission pathway under RCP8.5 scenario. The values are rounded off to the nearest 10s.

S.No.	States/UTs	2010	2030 (Baseline)	2030 (Mitigation)
1	Jammu and Kashmir	4600<br>(3750 – 5490)	8060<br>(5680 – 11000)	6020<br>(4240 - 8260)
2	UTs other than Delhi)	170<br>(130 – 210)	340<br>(220 - 510)	260<br>(170 -390)
3	Delhi	8630<br>(6970 – 9560)	14140<br>(10140 – 19190)	13440<br>(9600 - 18270)
4	Himachal Pradesh	3790<br>(3110 – 4490)	7200<br>(5110 – 9790)	5600<br>(3970 – 7590)
5	Haryana	32780<br>(27470 – 37930)	59860<br>(43470 – 79630)	53580<br>(39070 – 71560)
6	West Bengal	102,360<br>(85940 – 118,940)	176,330<br>(126,630 – 237,734)	140,990<br>(100,680 – 191,400)
7	Andhra Pradesh	59470<br>(44140 – 77840)	134,240<br>(87120 – 199,900)	106,420<br>(68260 – 159,520)
8	Kerala	14584<br>(11980 – 17240)	26010<br>(18370 – 35520)	19590<br>(13870 – 26700)
9	Orissa	35950<br>(28750 – 43910)	69540<br>(46890 – 99140)	57700<br>(38710 – 81940)
10	Karnataka	48010<br>(39670 – 56560)	93320<br>(67140 – 125,560)	72590<br>(51830 – 98010)
11	Maharashtra	68600<br>(56850 – 80260)	144,050<br>(104480 – 192,090)	116,700<br>(84780 – 156,490)
12	Assam	27320<br>(22490 – 32160)	45630<br>(32710 - 61670)	32460<br>(23100 – 44150)
13	Manipur	1860<br>(1450 – 2330)	3260<br>(2130 – 4780)	2370<br>(1540 – 3510)
14	Nagaland	1530<br>(1180 – 1950)	2680<br>(1700 - 4000)	1930<br>(1220 – 2890)
14	Meghalaya	2500<br>(1950 – 3140)	4200<br>(2720 - 6160)	2930<br>(1900 – 4320)
16	Punjab	25330<br>(21230 – 29360)	46220<br>(33850 – 61280)	37300<br>(27230 - 49600)
17	Rajasthan	50230<br>(41390 – 59260)	102,040<br>(72200 – 140,230)	80870<br>(57010 – 110,920)
18	Uttar Pradesh	225,230<br>(188,150 – 262,960)	410230<br>(293740 – 555,670)	335,310<br>(239430 – 455,820)
19	Uttaranchal	7490	13910	10480

		(6040 – 9030)	(9610 – 19450)	(7250 – 14640)
**20**	Jharkhand	40350<br>(33520 – 47390)	73920<br>(52310 – 101,380)	65580<br>(46310 – 89850)
**21**	Bihar	104,400<br>(87210 – 121,890)	177,620<br>(126,170 – 242,810)	137,680<br>(97400 – 188,410)
**22**	Sikkim	390<br>(300 – 490)	650<br>(430 – 960)	470<br>(300 - 690)
**23**	Chhattisgarh	27640<br>(22810 – 32540)	55730<br>(39920 – 75140)	46880<br>(33250 – 63660)
**24**	Madhya Pradesh	70010<br>(58070 – 82190)	142880<br>(102580 – 193800)	117,440<br>(83650 - 160150)
**25**	Tamil Nadu	44440<br>(36700 – 52420)	79280<br>(57510 – 106,490)	61980<br>(44680 – 83200)
**26**	Gujarat	38570<br>(31780 – 45280)	83110<br>(60020 – 111,560)	68220<br>(49180 – 91930)
**27**	Goa	350<br>(260 – 450)	740<br>(470 – 1120)	570<br>(360 – 860)
**28**	Arunachal Pradesh	1210<br>(950 – 1510)	2090<br>(1370 – 3010)	1510<br>(990 – 2200)
**29**	Mizoram	780<br>(600 – 970)	1370<br>(890 – 2000)	1020<br>(660 - 1500)
**30**	Tripura	2110<br>(1670 – 2600)	3720<br>(2480 – 5320)	2670<br>(1770 – 3850)
**31**	**India**	**1,050,340<br>(866,510 – 1,240,410)**	**1,982,370<br>(1,408,040 – 2,706,900)**	**1,600,580<br>(1,132,420 – 2,192,300)**
